# Fluorine-Modulated Reactivity Enables One-Pot Kinetic Sequence Programming of Block Copolyesters

**DOI:** 10.3390/polym18161977

**Published:** 2026-08-14

**Authors:** Chun-Yao Ke, Ryota Suzuki, Takuya Yamamoto, Takuya Isono, Guey-Sheng Liou, Toshifumi Satoh

**Affiliations:** 1 Institute of Polymer Science and Engineering, National Taiwan University, No. 1, Sec. 4, Roosevelt Road, Taipei 10617, Taiwan; chunyao.ke.m8@elms.hokudai.ac.jp; 2 Graduate School of Chemical Sciences and Engineering, Hokkaido University, Sapporo 060-8628, Japan; 3 Division of Applied Chemistry, Faculty of Engineering, Hokkaido University, Sapporo 060-8628, Japan; suzuki.r@eng.hokudai.ac.jp (R.S.); yamamoto.t@eng.hokudai.ac.jp (T.Y.); 4 List Sustainable Digital Transformation Catalyst Collaboration Research Platform, Institute for Chemical Reaction Design and Discovery, Hokkaido University, Sapporo 001-0021, Japan; 5 Department of Chemical and Materials Engineering, National Central University, No. 300, Zhongda Road, Zhongli District, Taoyuan 320317, Taiwan

**Keywords:** block copolymer, ring-opening alternating copolymerization, fluorinated polyesters, monomer reactivity, sequence control

## Abstract

Monomer sequence strongly influences copolymer properties, but direct block formation from a monomer mixture requires a large reactivity contrast. Here, fluorination was used to regulate anhydride reactivity in the cesium pivalate-catalyzed ring-opening alternating copolymerization (ROAC) of tetrafluorophthalic anhydride (FPA), phthalic anhydride (PA), and 3-perfluorohexyl-1,2-epoxypropane (PFE). Time-resolved nuclear magnetic resonance (NMR) spectroscopy showed that FPA reached >99% conversion before detectable PA incorporation. With 1,4-benzenedimethanol as a bidirectional initiator, this sequential consumption generated a central poly(FPA-*alt*-PFE) segment followed by poly(PA-*alt*-PFE) growth from both chain ends. Molar mass evolution, end-group analysis, and diffusion-ordered spectroscopy (DOSY) NMR collectively supported covalent block formation. Sequential incorporation was retained across three different FPA:PA feed ratios, and Beckingham–Sanoja–Lynd analysis yielded large and reciprocal effective reactivity-ratio descriptors ($r_{FPA}\approx 8.3–8.6\times 10^{2}$ and $r_{PA}\approx 1.2\times 10^{-3}$), consistent with the experimentally observed real-block regime. Matched model reactions further indicated faster FPA ring-opening and higher observed epoxide-opening reactivity in a fluorinated aromatic carboxylate model system. These results demonstrate that H-to-F substitution can provide the kinetic bias required to program block copolyester sequence within a single ROAC platform.

## 1. Introduction

Block copolymers (BCPs) covalently link chemically distinct polymer segments, allowing combinations of properties that are not readily obtained from homopolymers or physical blends. Because the segments are tethered to one another, macrophase separation is suppressed and, when the blocks are sufficiently incompatible, organization into nanoscopic domains becomes possible. The resulting morphology depends on block composition, molar mass, architecture, and intersegment interactions [1,2]. This structural control has supported applications in nanolithography [3,4,5,6], selective membranes and porous materials [7,8,9,10,11,12,13,14], biomedical systems [15,16,17,18], and solid polymer electrolytes [19,20,21,22]. The preparation of BCPs therefore requires control not only over segment chemistry but also over the order in which the segments are formed.

BCPs are commonly prepared by sequential monomer addition [23,24,25,26], orthogonal or sequential combinations of polymerization methods using dual-functional initiators [27,28,29,30], chain growth from telechelic macroinitiators or macro-chain-transfer agents [31,32,33,34], or coupling of separately prepared end-functional polymers [35,36,37,38]. These approaches are versatile, but block order is imposed through repeated external operations. Monomer feeding, intermediate isolation, chain-end conversion, and polymer coupling increase the number of synthetic steps, while incomplete reinitiation or coupling can leave residual precursor polymers. These difficulties become increasingly significant as more segments are introduced.

**Figure 1:**
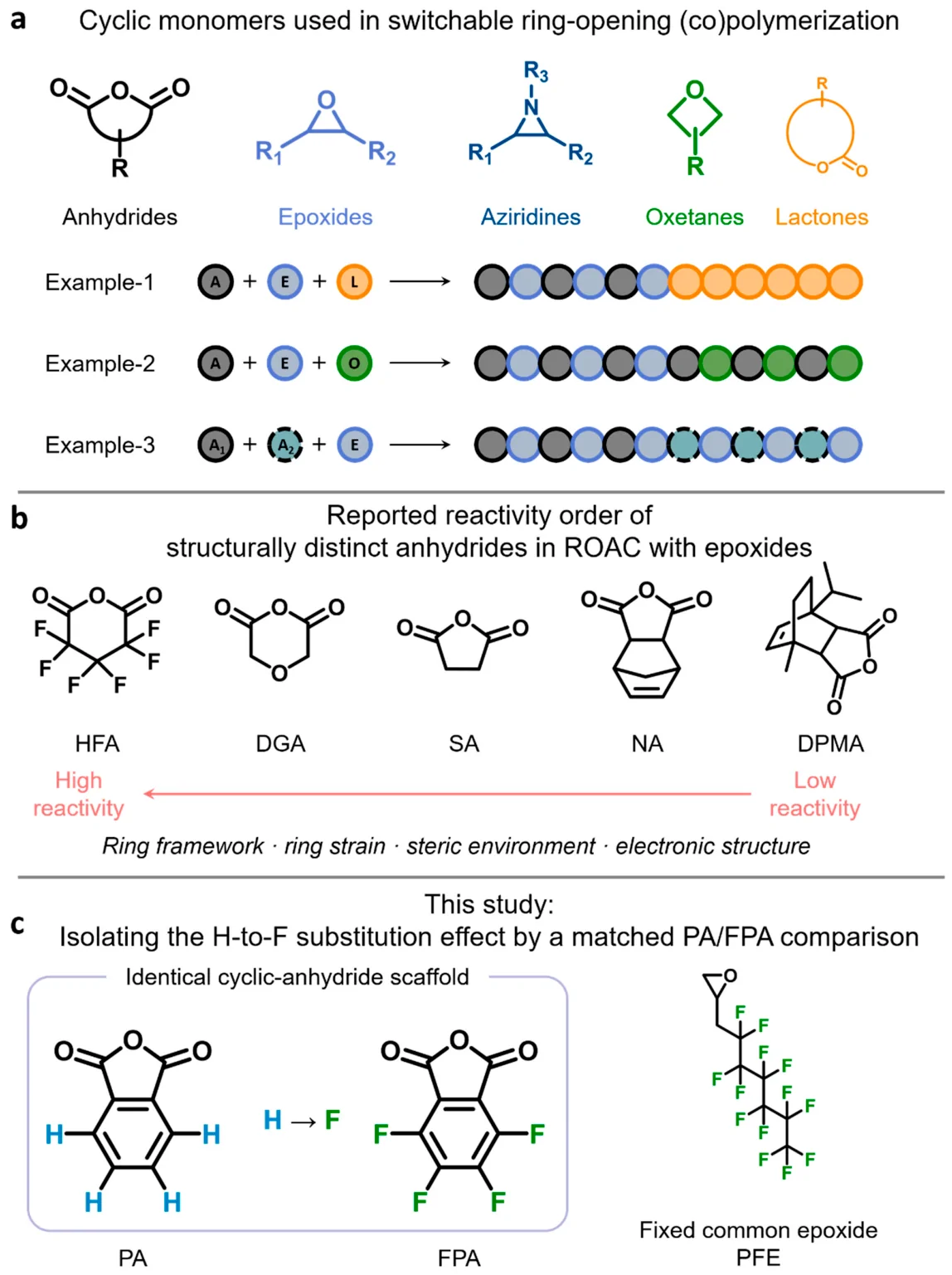
Conceptual framework for isolating the effect of anhydride fluorination in sequence-controlled ring-opening alternating copolymerization. (**a**) Representative cyclic monomers used in switchable and self-switchable ring-opening (co)polymerizations, together with schematic examples of anhydride/epoxide ROAC followed by lactone ROP, anhydride/oxetane ROAC, or ROAC with a second anhydride [39,40,41,42,43,44,45]. A, E, L, and O denote anhydride, epoxide, lactone, and oxetane, respectively. (**b**) Literature-reported reactivity order of structurally distinct cyclic anhydrides in ROAC with epoxides: HFA ≫ DGA > SA > NA ≫ DPMA [44]. Their simultaneous differences in ring framework, ring strain, steric environment, and electronic structure prevent isolation of an individual structural effect. (**c**) Matched PA/FPA comparison using PFE as the common epoxide to isolate the effect of H-to-F substitution; ROAC of PFE with both anhydrides was established previously [48]. HFA, 2,2,3,3,4,4-hexafluoropentanedioic anhydride; DGA, diglycolic anhydride; SA, succinic anhydride; NA, nadic anhydride; DPMA, dipentene–maleic anhydride. ROAC, ring-opening alternating copolymerization; ROP, ring-opening polymerization; PA, phthalic anhydride; FPA, tetrafluorophthalic anhydride; PFE, 3-perfluorohexyl-1,2-epoxypropane.

One-pot polymerization from a monomer mixture offers a different approach. When competing monomers or polymerization cycles have sufficiently different reactivities, the more favorable combination is consumed first and the remaining monomers are incorporated at a later stage. Provided that the chain ends retain their activity, block polymers can be formed without sequential feeding or post-polymerization coupling. This principle has been used in switchable and self-switchable polymerizations involving cyclic anhydrides, epoxides, oxetanes, lactones, and aziridines (Figure 1a) [39,40,41,42,43,44,45]. Differences in monomer class, ring-opening kinetics, and catalytic-cycle selectivity have enabled block polyesters with increasingly complex sequences and architectures.

Within alkali-metal-carboxylate-catalyzed ring-opening alternating copolymerization (ROAC), anhydride and epoxide insertion play different kinetic roles. Selection between competing anhydrides occurs during alkoxide-mediated anhydride opening, whereas epoxide opening by the resulting carboxylate is slower and is generally regarded as the rate-determining propagation step [41,42,43,44,46]. The anhydride structure can therefore affect both its competitive incorporation and the reactivity of the carboxylate chain end generated after ring opening. Reactivity hierarchies have been established for a range of cyclic anhydrides and quantified using the Beckingham-Sanoja-Lynd nonterminal model [44,47]. Depending on the magnitude of the effective reactivity ratios, competitive ROAC can yield gradient, block-like, or real-block sequences.

Previous studies have related anhydride reactivity to several structural factors. For example, a substituted bicyclic anhydride was located at the low-reactivity end of the reported hierarchy, whereas side-chain substitution within the succinic anhydride series produced only comparatively small changes in reactivity [42,44]. A correlation between anhydride reactivity and the ^13^C nuclear magnetic resonance (NMR) chemical shifts in the carbonyl carbons was observed, but carbonyl electron density alone did not reproduce the experimental order [44]. The reported trends were therefore interpreted as the combined outcome of ring strain, steric accessibility, and electronic structure. Because the compared anhydrides differed in several structural features simultaneously, the contribution of each factor could not be isolated (Figure 1b).

A particularly large reactivity contrast was observed between 2,2,3,3,4,4-hexafluoropentanedioic anhydride (HFA) and diglycolic anhydride (DGA), allowing a real-block copolymer to form from a mixed monomer feed [44]. This result suggested that fluorination may contribute strongly to anhydride selectivity. However, the two monomers differ not only in fluorine content but also in carbon skeleton, heteroatom content, ring environment, and the structures of their corresponding carboxylate chain ends. Consequently, the role of fluorination itself remains difficult to distinguish from other structural effects.

Phthalic anhydride (PA) and tetrafluorophthalic anhydride (FPA) provide a more direct comparison (Figure 1c). They share the same aromatic cyclic-anhydride scaffold, while the four aromatic hydrogen atoms in PA are replaced by fluorine atoms in FPA. This matched pair minimizes differences in ring framework and structural rigidity, allowing the overall effect of H-to-F substitution to be evaluated within a common ROAC framework. This substitution may influence carbonyl electrophilicity, ion pairing, chain-end reactivity, solubility, aggregation, and local interactions with the highly fluorinated 3-perfluorohexyl-1,2-epoxypropane (PFE) reaction medium. It therefore provides a means of testing whether the combined consequences of anhydride fluorination can generate the kinetic contrast required for block formation. Cesium pivalate (CsOPiv) has previously enabled ROAC of related fluorinated anhydrides and perfluoroalkyl-substituted epoxides [48], providing a suitable catalytic platform for this comparison.

Because PFE had previously been shown to undergo ROAC with both FPA and PA [48], it was selected as the common epoxide to examine the effect of anhydride fluorination while keeping the epoxide-derived repeat unit constant. The three monomers were accordingly subjected to one-pot terpolymerization using CsOPiv and 1,4-benzenedimethanol (BDM) as the catalyst and bidirectional initiator, respectively. FPA was consumed before detectable PA incorporation, producing a central poly(FPA-*alt*-PFE) segment before poly(PA-*alt*-PFE) growth proceeded from both chain ends. Time-resolved ^1^H and ^19^F NMR spectroscopy, size-exclusion chromatography (SEC), molar-mass evolution, and diffusion-ordered NMR spectroscopy were used to follow the sequential chain-growth process and evaluate covalent block formation. Effective reactivity ratios were obtained from compositional-drift data using the Beckingham-Sanoja-Lynd nonterminal model, and matched model reactions were used to examine how fluorination affects monomer and chain-end reactivity. The results show that H-to-F substitution can provide the kinetic bias required to program block formation within a single anhydride/epoxide ROAC cycle.

## 2. Materials and Methods

### 2.1. Materials

Unless otherwise stated, all chemicals were used as received. Isopropyl alcohol (IPA; >99.5%, Tokyo Chemical Industry Co., Ltd. (TCI), Tokyo, Japan), 1,1,1-trifluoro-2-propanol (TrFPA; >97.0%, TCI), 3-perfluorohexyl-1,2-epoxypropane (PFE; >98%, TCI), 3-phenyl-1-propanol (PPA; >98.0%, TCI), propylene oxide (PO; >99.0%, TCI), 1,2-epoxydecane (DO; >97.0%, TCI), and 3,3,3-trifluoropropylene oxide (TrFE; >98%, TCI) were distilled over CaH_2_ under reduced pressure and stored under nitrogen. Phthalic anhydride (PA; >98.0%, TCI) and tetrafluorophthalic anhydride (FPA; >95.0%, TCI) were recrystallized and purified by sublimation. Cesium pivalate (CsOPiv; >97.0%, TCI), benzoic acid (BzA; >99.0%, TCI), 2,3,4,5-tetrafluorobenzoic acid (FBzA; >98.0%, TCI), and 1,4-benzenedimethanol (BDM; >99.0%, TCI) were dried at 100 °C under high vacuum for at least 72 h. All air- and moisture-sensitive manipulations were performed under argon using standard glovebox or Schlenk techniques.

### 2.2. Characterization

^1^H and ^19^F NMR spectra were recorded on a JEOL JNM-A400II spectrometer (JEOL Ltd., Tokyo, Japan) using CDCl_3_ or tetrahydrofuran-d_8_ (THF-d_8_) as the solvent at 25 °C. Spectra used for conversion and end-group analysis were acquired using a 45° pulse, eight scans, and a relaxation delay of 5 s. The spectra were processed in MestReNova 14.0.0, and phase and baseline corrections were applied before manual integration. Consistent integration regions were used within each time series. Chemical structures and reaction schemes were prepared using ChemDraw Professional 15.0. Monomer conversions were calculated from the relative integrals of the residual monomer and corresponding polymer resonances. BDM-based segment degrees of polymerization (DPs) were calculated from the repeat-unit-to-BDM integral ratios after correction for the respective numbers of nuclei. Because of the low relative intensity of the BDM resonances, the resulting DPs and *M_n,NMR_* values were treated as approximate estimates. Diffusion-ordered NMR spectroscopy (DOSY) was performed at 30 °C using the ledbpgp2s pulse sequence with at least 15 gradient increments.

**Figure 2:**
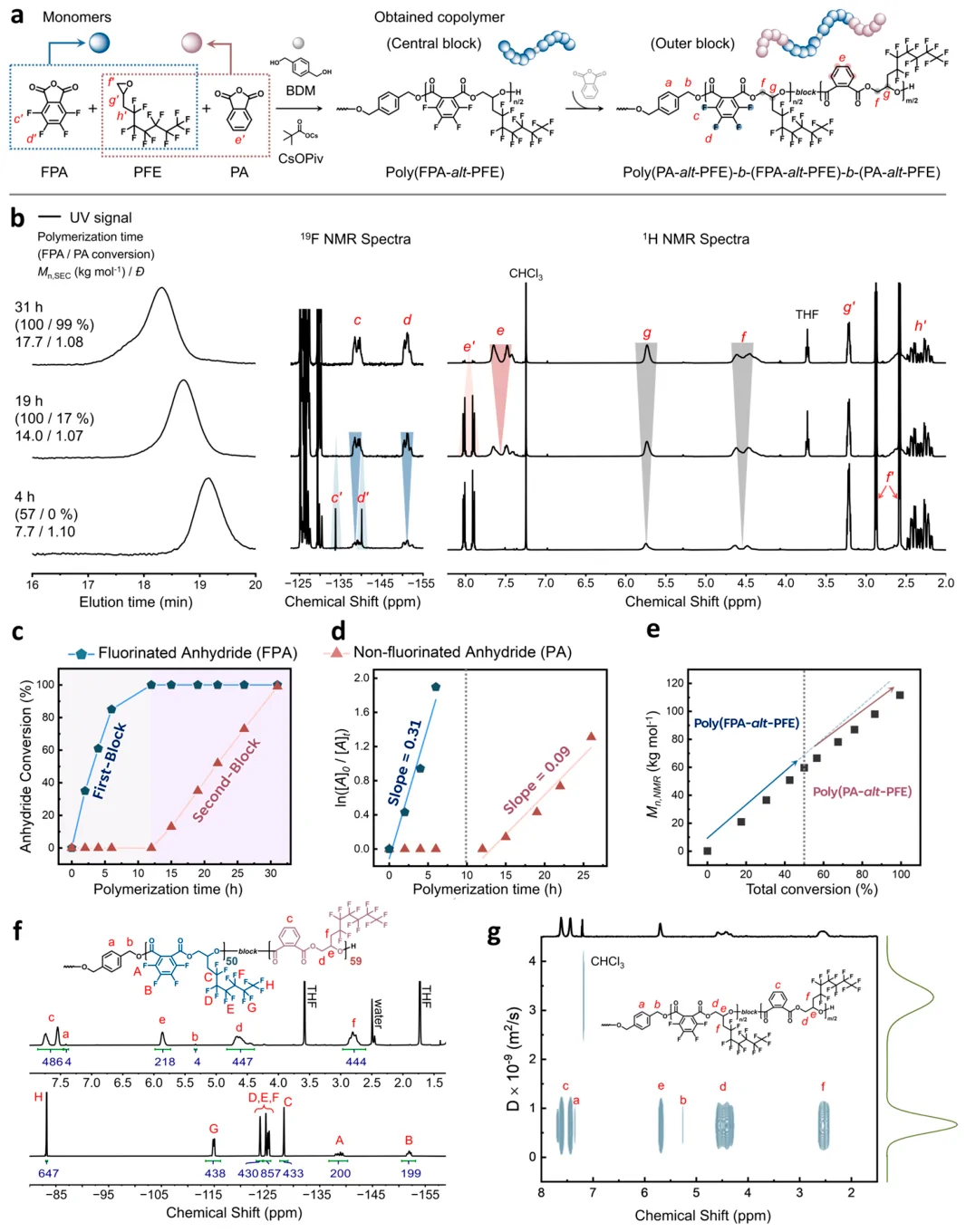
One-pot ROAC of FPA, PA, and PFE under CsOPiv catalysis. (**a**) Synthetic route and triblock architecture. (**b**) Time-resolved SEC traces (THF eluent; UV detection at 280 nm), ^19^F NMR spectra, and ^1^H NMR spectra. (**c**) Anhydride conversion versus time. (**d**) Plot used to describe the FPA- and PA-dominated consumption regimes. (**e**) *M_n,NMR_* versus total anhydride conversion. (**f**) Representative ^1^H and ^19^F NMR spectra of purified F100–N100 in THF-d_8_; lowercase/uppercase labels denote ^1^H/^19^F resonances, respectively. (**g**) DOSY NMR spectrum in CDCl_3_. Abbreviations: ROAC, ring-opening alternating copolymerization; FPA, tetrafluorophthalic anhydride; PA, phthalic anhydride; PFE, 3-perfluorohexyl-1,2-epoxypropane; CsOPiv, cesium pivalate; BDM, 1,4-benzenedimethanol; SEC, size-exclusion chromatography; THF, tetrahydrofuran; UV, ultraviolet; NMR, nuclear magnetic resonance; DOSY, diffusion-ordered spectroscopy.

SEC measurements were performed at 40 °C and 1.0 mL min^−1^ using a JASCO high-performance liquid chromatography (HPLC) system (JASCO Corporation, Hachioji, Japan) equipped with a PU-4180 pump, an AS-4550 autosampler, a CO-4060 column oven, a Shodex K-800D guard column (Resonac Corporation, Tokyo, Japan), and Shodex K-806L and K-804L columns. THF was used as the eluent for the time-resolved SEC measurements shown in Figure 2, Figure S1 and S2. The chromatograms were recorded using an ultraviolet (UV) absorbance detector at 280 nm because the fluorinated polymers produced no analytically useful response under conventional refractive-index (RI) detection in THF. Number-average molar masses (*M*_n,SEC_) and dispersities (*Đ*) were determined using polystyrene standards (*M*_n_ = 1200–1,320,000 g mol^−1^).

Preparative SEC was carried out at 25 °C in CHCl_3_ with a flow rate of 10.0 mL min^−1^ using a LaboACE LC-7080 system (Japan Analytical Industry Co., Ltd., Tokyo, Japan) equipped with a JAIGEL-HR-P guard column (8 mm × 40 mm), a JAIGEL 2HR column (20.0 mm × 600 mm; exclusion limit, 5.0 × 10^3^), and a JAIGEL 2.5HR column (20.0 mm × 600 mm; exclusion limit, 2.0 × 10^4^).

For absolute molar-mass determination, the same JASCO HPLC system and column set were coupled to a DAWN 8 multiangle light scattering (MALS) detector (Wyatt Technology, Santa Barbara, CA, USA), a Viscostar viscosity detector (Wyatt Technology), and an RI-501 refractive index detector (Shodex). SEC-MALS-Visco measurements were performed in CHCl_3_ at 40 °C with a flow rate of 1.0 mL min^−1^. The absolute number-average molar mass (*M_n,MALS_*), dispersity (*Đ_MALS_*), and dn/dc values were determined from the MALS and refractive-index data.

Differential scanning calorimetry (DSC) measurements were performed using a Hitachi High-Tech Science DSC 7000X instrument (Hitachi High-Tech Science Corporation, Tokyo, Japan) under a nitrogen atmosphere. Samples were first heated from room temperature to 250 °C, held at 250 °C for 10 min to erase their thermal history, cooled to −100 °C, and then reheated to 250 °C at a rate of 10 °C min^−1^. Glass-transition temperatures were determined from the second heating traces. Thermogravimetric analysis (TGA) was performed using a Hitachi High-Tech Science STA200RV instrument (Hitachi High-Tech Science Corporation, Tokyo, Japan) under nitrogen from 30 to 600 °C at a heating rate of 10 °C min^−1^.

### 2.3. Representative Polymerization Procedure

The sample notation “Fm–Nn” denotes the targeted numbers of FPA-derived (F) and PA-derived (N) alternating repeat units per BDM initiator; both segments contain PFE-derived units. For F100–N100, CsOPiv (11.70 mg, 0.05 mmol, 0.5 equiv), BDM (13.92 mg, 0.10 mmol, 1.0 equiv), FPA (2200.8 mg, 10.0 mmol, 100 equiv), PA (1481.2 mg, 10.0 mmol, 100 equiv), and PFE (15,044.8 mg, 40.0 mmol, 400 equiv) were charged into a predried reaction vessel equipped with a magnetic stir bar in an argon-filled glovebox. The mixture was heated at 120 °C; excess PFE served as both comonomer and reaction medium. Aliquots were withdrawn periodically and immediately cooled to room temperature. Aliquots intended for ^1^H/^19^F NMR analysis were diluted with CDCl_3_, whereas those intended for SEC analysis were diluted with THF. Rapid cooling from 120 °C to room temperature, together with substantial dilution, effectively halted further propagation without requiring an additional chemical terminating reagent. FPA conversion was determined from the shift in its ^19^F resonances from approximately −133 and −140 ppm to −139 and −151 ppm, respectively; PA conversion was determined from the shift in its aromatic ^1^H resonances from 7.9–8.0 to 7.5–7.6 ppm. At approximately 12 h, the FPA conversion exceeded 99%, while PA remained unreacted. Although the reaction mixture remained homogeneous at 120 °C, its viscosity increased substantially after formation of the poly(FPA-*alt*-PFE) segment. Dry THF (1.0 mL) was therefore added to maintain effective stirring during the subsequent PA–PFE growth stage. The polymer-rich mixture became heterogeneous only upon cooling. After completion, the mixture was diluted with THF and poured into cold methanol. The polymer was purified by preparative SEC to remove residual epoxide and catalyst, then dried under vacuum at 80 °C to afford F100–N100 as a white solid (10.66 g, 95% isolated yield).

### 2.4. Analogous Polymerizations for F50-N150 and F150-N50

F50–N150 was prepared analogously using CsOPiv (11.70 mg, 0.05 mmol), BDM (13.92 mg, 0.10 mmol), FPA (1100.4 mg, 5.0 mmol), PA (2221.8 mg, 15.0 mmol), and PFE (15,044.8 mg, 40.0 mmol), and was isolated in 95% yield. F150–N50 was prepared using CsOPiv (11.70 mg, 0.05 mmol), BDM (13.92 mg, 0.10 mmol), FPA (3301.2 mg, 15.0 mmol), PA (740.6 mg, 5.0 mmol), and PFE (15,044.8 mg, 40.0 mmol), and was isolated in 93% yield. For F150–N50 and F50–N150, dry THF (1.0 mL) was added at approximately 4.5 and 4 h, respectively, after FPA conversion exceeded 99%; the corresponding PA conversions were approximately 0% and 14%, respectively. The polymerization conditions, final conversions, molar masses, dispersities, and isolated yields for F100–N100, F150–N50, and F50–N150 are summarized in Table S2. The corresponding reagent charges are compiled in Table S3.

### 2.5. Determination of Effective Reactivity Ratios Using the BSL Nonterminal Model

Effective reactivity ratios were determined from compositional-drift data using the Beckingham–Sanoja–Lynd (BSL) nonterminal model [47]. PA and FPA were designated *A*_1_ and *A*_2_*,* respectively. The individual anhydride conversions and overall conversion were defined as:

$$p_{FPA}=1-\frac{{FPA}_{t}}{{FPA}_{0}}$$

$$p_{PA}=1-\frac{{PA}_{t}}{{PA}_{0}}$$

$$p_{total}=1-\frac{\left({FPA}_{t}+{PA}_{t}\right)}{\left({FPA}_{0}+{PA}_{0}\right)}=n_{FPA}\times p_{FPA}+n_{PA}\times p_{PA}$$

where $n_{FPA}$ and $n_{PA}$ denote the initial mole fractions of FPA and PA, respectively. Under the BSL nonterminal model, the monomer-consumption relationships are expressed as:

$$1-p_{FPA}={\left(1-p_{PA}\right)}^{r_{FPA}}$$

$$1-p_{PA}={\left(1-p_{FPA}\right)}^{r_{PA}}$$

Substitution into the overall-conversion expression gives:

$$p_{total}=1-n_{FPA}\left(1-p_{FPA}\right)-{n_{PA}\left(1-p_{FPA}\right)}^{r_{PA}}$$

$$p_{total}=1-{n_{FPA}\left(1-p_{PA}\right)}^{r_{FPA}}-n_{PA}\left(1-p_{PA}\right)$$

The compositional-drift data obtained at initial FPA:PA feed ratios of 100:100, 150:50, and 50:150 were fitted independently to these expressions by nonlinear least squares. In the strict nonterminal limit, $r_{FPA}r_{PA}=1$; however, the two parameters were fitted independently to accommodate experimental noise. All measured compositional-drift data points were included in the fitting, and no outlier rejection or other data exclusion was applied.

### 2.6. Model Reactions

All model reactions were conducted using CsOPiv as the catalyst in anhydrous THF. Unless otherwise noted, the two organic substrates and CsOPiv were combined at a nominal molar ratio of 1:1:0.2, with each organic substrate at an initial concentration of approximately 1.0 M. The reactions were performed in predried, sealed vessels under argon at 60 or 100 °C, as specified for each reaction.

As a representative procedure, IPA, PA, and CsOPiv were combined in anhydrous THF under the conditions described above, and the reaction mixture was stirred at 60 °C. At predetermined time points, aliquots were withdrawn under argon, immediately diluted with CDCl_3_, and analyzed by ^1^H NMR spectroscopy. The remaining model reactions were conducted using the same general handling and sampling procedures, with the substrate combinations and reaction temperatures specified for each reaction. The identities and quantities of the reagents and the solvent volumes used for the individual model reactions are summarized in Table S4.

Conversions were determined from the relative integrations of diagnostic resonances assigned to the cyclic substrates and the corresponding ring-opened products. The conversion equations and additional data-treatment details are provided in Section S2 of the Supporting Information.

## 3. Results and Discussion

### 3.1. Competitive Terpolymerization Behavior of FPA, PA, and PFE

FPA and PA were selected as a matched aromatic anhydride pair to test whether H-to-F substitution, used as the controlled molecular modification, could create sufficient reactivity contrast for block formation. PFE was used as the common epoxide, and BDM initiated growth from two hydroxyl groups so that the first-formed segment would occupy the chain center (Figure 2a). At [BDM]_0_/[CsOPiv]_0_/[FPA]_0_/[PA]_0_/[PFE]_0_ = 1:0.5:100:100:400, time-resolved ^19^F and ^1^H NMR spectra showed temporally separated anhydride consumption (Figure 2b). The FPA resonances at −133 and −140 ppm shifted quantitatively to −139 and −151 ppm during the first stage, whereas the PA aromatic signals at 7.9–8.0 ppm remained unchanged. Only after FPA depletion did the PA signals shift to 7.5–7.6 ppm, indicating growth of the PA-derived outer segments.

The conversion profiles confirm that FPA reached >99% conversion before detectable PA consumption (Figure 2c). Over the same period, the SEC distribution remained unimodal and shifted to shorter elution time: *M_n,SEC_* increased from 7.7 kg mol^−1^ at 4 h (FPA/PA conversion = 57/0%; *Đ* = 1.10) to 14.0 kg mol^−1^ at 19 h (100/17%; *Đ* = 1.07) and 17.7 kg mol^−1^ at 31 h (100/99%; *Đ* = 1.08) (Figure 2b). These changes are consistent with chain extension of the first-formed poly(FPA-*alt*-PFE) segment and show no resolved precursor population. For descriptive comparison within each conversion regime, linear fits of the corresponding semilogarithmic plots gave apparent slopes of 0.31 and 0.09 h^−1^ for the FPA- and PA-dominated stages, respectively (Figure 2d). Because the reaction composition and physical state differed between the two stages, these slopes are used only to describe the observed consumption profiles and are not interpreted as intrinsic or directly comparable rate constants. The preferential consumption of FPA was already evident before THF addition and therefore cannot be attributed to the subsequent solvent change. The reaction mixture remained homogeneous at 120 °C but became highly viscous after formation of the poly(FPA-*alt*-PFE) segment. Dry THF (1.0 mL) was added only at this stage to maintain effective stirring during PA-derived growth; precipitation was observed only upon cooling. Preliminary trials with THF present from the beginning were feasible at a target DP of 25 but markedly retarded polymerization at a target DP of 100, leaving PA incompletely converted even after approximately one month. The staged bulk-to-solution protocol was therefore selected to maintain a sufficiently high initial monomer concentration while minimizing solvent use.

Sustained chain-end activity was further supported by the approximately linear increase in $M_{n,NMR}$ with conversion in two regimes (Figure 2e). Because the two hydroxyl groups of BDM are chemically equivalent, the NMR-derived total segment DPs were expressed as nominal per-arm values assuming statistically comparable bidirectional initiation and growth. On this basis, F100–N100 contained average per-arm DPs of approximately 50 FPA-derived and 59 PA-derived repeat units, corresponding to total segment DPs of approximately 100 and 118 per BDM-initiated chain and an FPA-derived/PA-derived composition of approximately 46:54 mol%. These values represent ensemble averages and do not imply exact equality of the two arms in every polymer molecule. These values indicate that the two segment types had comparable overall lengths, with a slightly higher PA-derived contribution. The FPA contribution was independently checked by ^19^F NMR integration (Figure 2f). The time-resolved SEC chromatograms of F100–N100 showed a continuous shift in the same unimodal polymer population toward higher molecular weight during the transition from FPA consumption to PA-derived growth, providing the primary evidence for chain extension rather than the formation of separate polymers. DOSY NMR showed a single self-diffusion coefficient for all polymer-associated proton resonances (Figure 2g). Because the FPA-derived segment does not contain proton resonances that can be uniquely distinguished from those of the PA-derived segment, the DOSY result is treated as supporting, rather than standalone, evidence that the polymer signals arise from a single molecular entity in solution. Together with the continuous SEC chain-extension behavior, sequential FPA-to-PA consumption, and BDM end-group analysis, these results provide strong evidence for covalent formation of poly(PA-*alt*-PFE)-*b*-poly(FPA-*alt*-PFE)-*b*-poly(PA-*alt*-PFE), rather than a physical blend of independently formed polymers. The same switch-like incorporation was retained for both F150–N50 and F50–N150. The SEC distributions of F150–N50 remained unimodal throughout the reaction, whereas the principal SEC elution peak of F50–N150 shifted toward higher molar mass but developed a minor shoulder on the low-molar-mass side at high conversion (Figures S1 and S2). The two samples were prepared at initial [BDM]_0_/[CsOPiv]_0_/[FPA]_0_/[PA]_0_/[PFE]_0_ ratios of 1:0.5:150:50:400 and 1:0.5:50:150:400, respectively. F150–N50 exhibited an $M_{n,SEC}$ of 12.0 kg mol^−1^ and a *Đ* of 1.08, and end-group analysis gave approximately 74 FPA-derived and 26 PA-derived repeat units per arm, corresponding to an FPA-derived/PA-derived composition of approximately 74:26 mol%. F50–N150 exhibited $M_{n,SEC}$ of 49.7 kg mol^−1^ and *Đ* of 1.12, with approximately 20 FPA-derived and 70 PA-derived repeat units per arm, corresponding to a composition of approximately 22:78 mol%. The measured segment compositions were consistent with the respective targeted ratios. Thus, the block ratio can be varied without changing the monomer set or introducing a second monomer feed, although the PA-rich formulation showed somewhat less uniform molar-mass evolution.

Absolute molar masses were further determined by SEC-MALS in CHCl_3_ (Figure S3). F100–N100, F150–N50, and F50–N150 exhibited *M_n,MALS_* values of 91.1, 99.5, and 92.8 kg mol^−1^, respectively, in reasonable agreement with their NMR-estimated and theoretical molar masses. These results confirm that the actual molar masses of all three samples are close to 100 kg mol^−1^. In contrast, the corresponding polystyrene-calibrated *M_n,SEC_* values were 15.9, 11.6, and 23.6 kg mol^−1^. The lower apparent SEC values result from differences in hydrodynamic volume between the fluorinated polyesters and polystyrene standards. Their composition dependence reflects changes in solvation and chain conformation with the FPA:PA composition, rather than differences in absolute molar mass.

### 3.2. Effective Reactivity-Ratio Analysis

**Figure 3:**
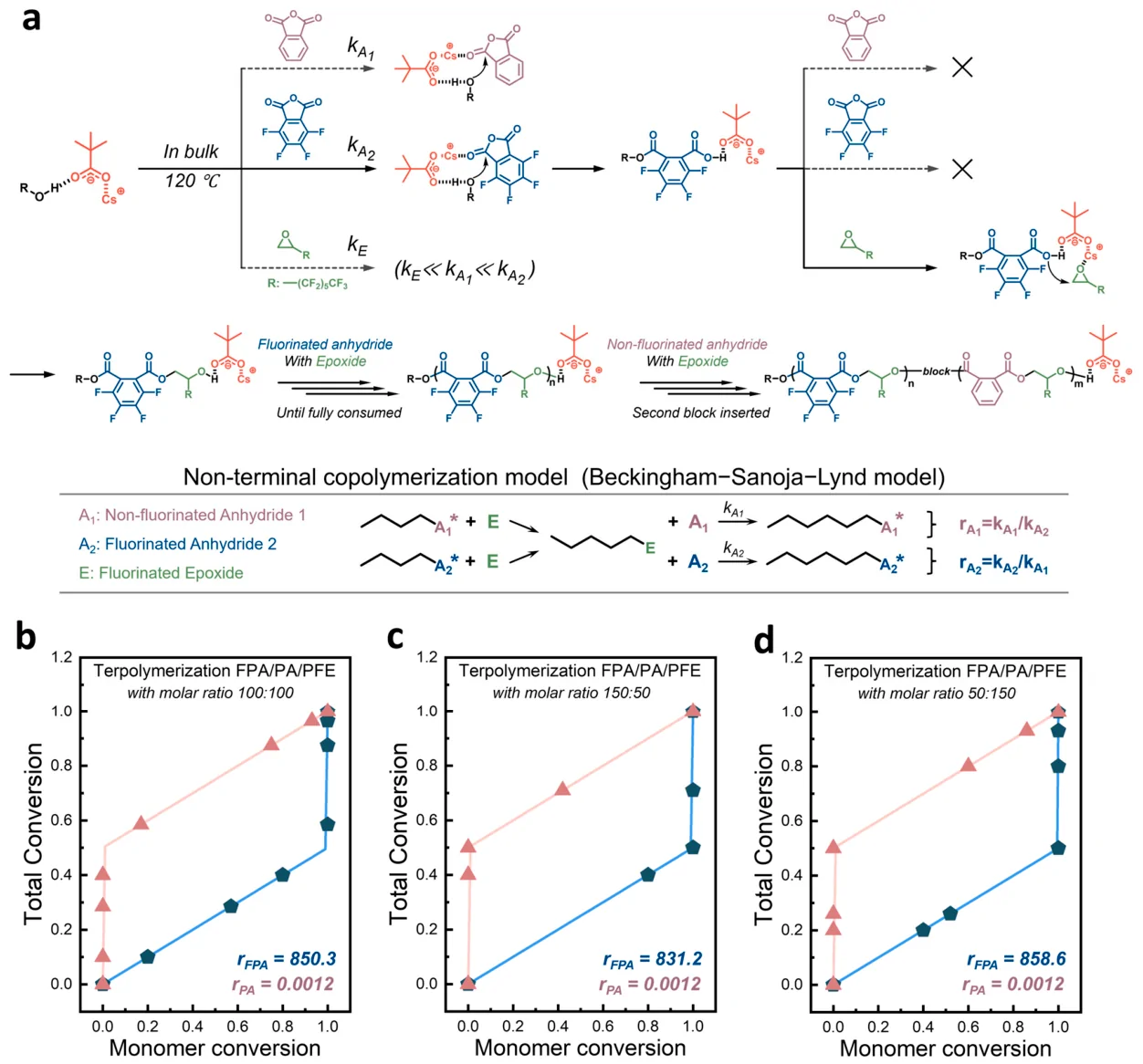
Quantitative analysis of competitive anhydride incorporation. (**a**) Schematic representation of the one-pot sequence and BSL nonterminal model. PA is A_1_ and FPA is A_2_; therefore, *r*_PA_ = *k*_A1_/*k*_A2_ and *r*_FPA_ = *k*_A2_/*k*_A1_. (**b**–**d**) Overall anhydride conversion versus individual anhydride conversion for FPA:PA feeds of (**b**) 100:100, (**c**) 150:50, and (**d**) 50:150. BSL, Beckingham–Sanoja–Lynd.

The magnitude of the anhydride selectivity was quantified using the BSL nonterminal model (Figure 3; Section 2.5) [47]. The fitted effective $r_{FPA}$ values for F100–N100, F150–N50, and F50–N150 were 850.3, 831.2, and 858.6, respectively, whereas $r_{PA}$ remained approximately $1.2\times 10^{-3}$ for all three feed compositions. All three systems were therefore classified in the real-block regime (Figure 3b–d; Table S1). Because PA incorporation was strongly suppressed during the FPA-dominated stage, these fitted values are interpreted primarily in terms of their magnitude and sequence classification rather than as precisely determined elementary rate constants. The fitted effective reactivity ratios, their products, and the corresponding sequence classifications are summarized in Table S1. The agreement across feed compositions supports the internal consistency of the effective nonterminal treatment and places all three mixtures in the real-block regime. The fitted ratios consistently describe the extreme compositional drift characteristic of the real-block regime. They therefore provide a quantitative description of the suppression of PA incorporation while FPA remains present.

### 3.3. Matched Model Reactions and Mechanistic Interpretation

**Figure 4:**
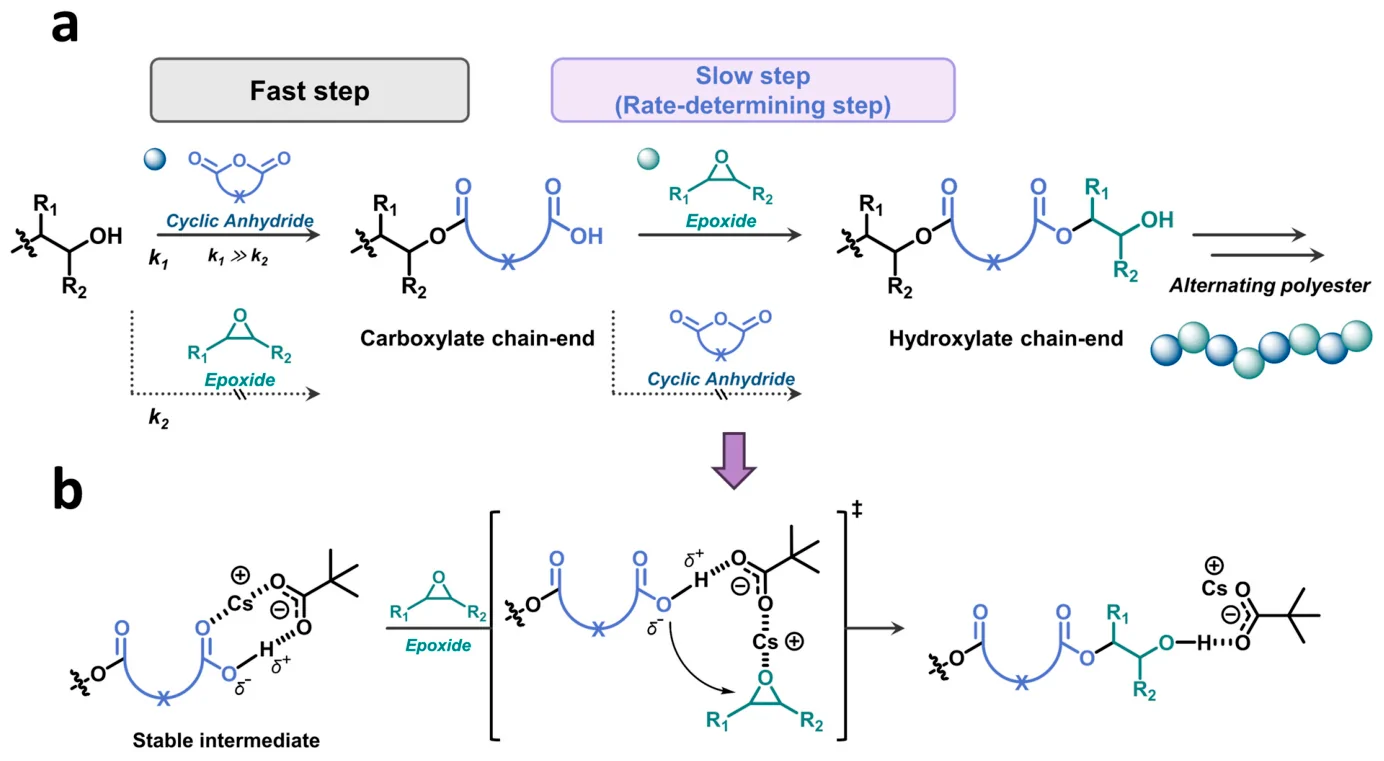
Working mechanistic framework for anionic ROAC. (**a**) Alternation between alkoxide-mediated anhydride opening and the slower carboxylate-mediated epoxide-opening step. (**b**) Proposed ion-paired cesium carboxylate resting state; the energy-well representation is qualitative.

The BSL analysis describes the net kinetic bias but does not identify which ROAC step is altered by fluorination. Within the commonly proposed alkali-metal-carboxylate mechanism, alkoxide-mediated anhydride opening is rapid, whereas epoxide opening by the resulting carboxylate is slower and commonly rate-determining [41,44,46]. The carboxylate may also populate an ion-paired cesium resting state (Figure 4). Within this working model, fluorination may delocalize the anionic charge and reduce the localized charge density at the carboxylate oxygen, making the specific Cs^+^–carboxylate association less persistent and facilitating reorganization toward epoxide opening. Fluorination could therefore influence both selection of the incoming anhydride and the reactivity of the chain end formed after its incorporation. This interpretation remains qualitative.

**Figure 5:**
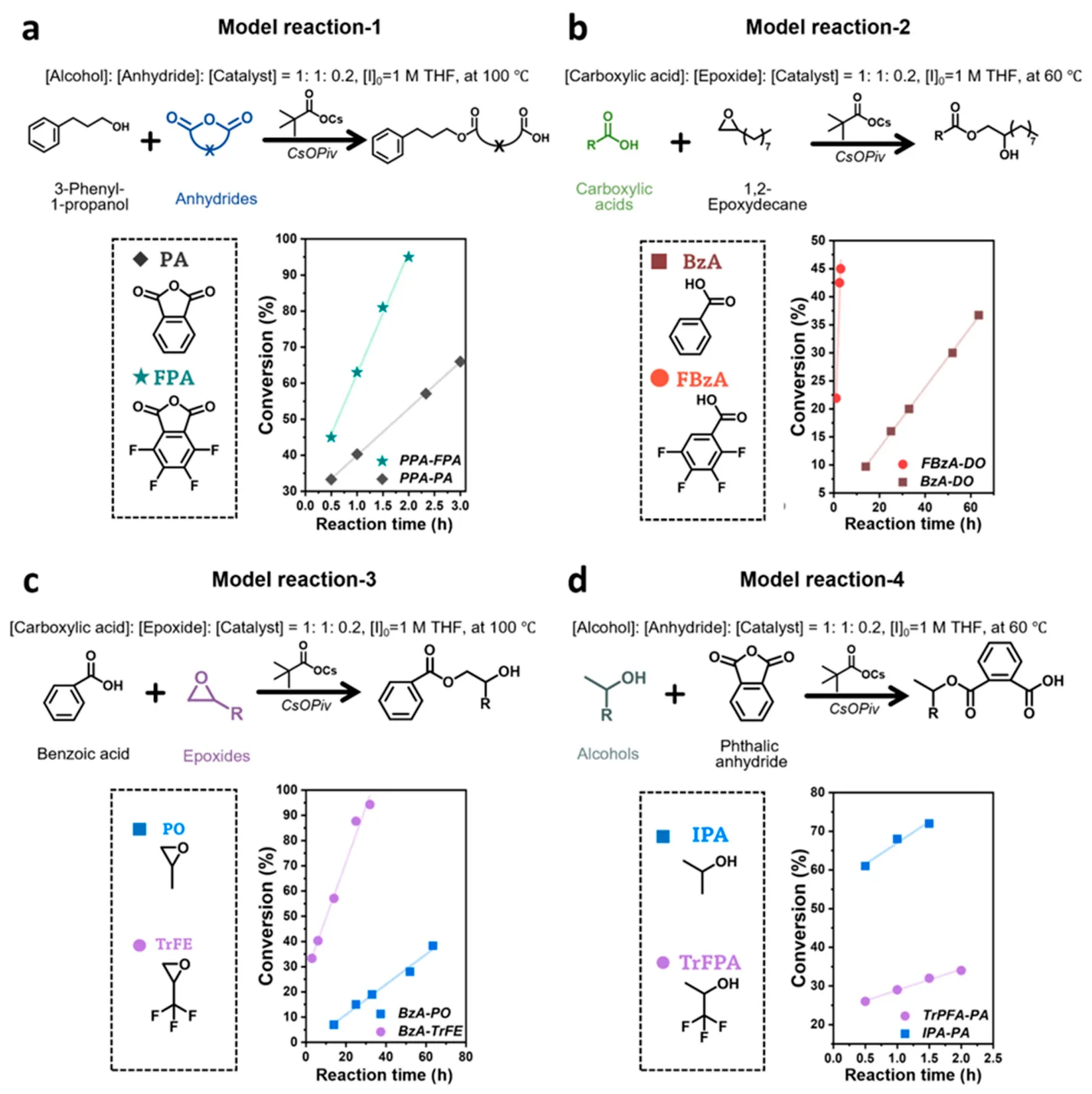
Matched model reactions comparing fluorinated and non-fluorinated analogs under CsOPiv catalysis. Reactions were conducted in THF under argon using substrate/substrate/CsOPiv = 1:1:0.2, with each organic substrate at an initial concentration of 1.0 M, at 60 or 100 °C as indicated. Conversion was determined by time-resolved NMR spectroscopy. (**a**) Alcohol-mediated opening of PA versus FPA. (**b**) DO opening in the presence of BzA versus FBzA carboxylic acids. (**c**) BzA-mediated opening of PO versus TrFE. (**d**) PA opening by IPA versus TrFPA alcohol/alkoxide surrogates. All measured points are shown; the lines are visual guides and do not represent kinetic fits. Abbreviations: PPA, 3-phenyl-1-propanol; DO, 1,2-epoxydecane; BzA, benzoic acid; FBzA, 2,3,4,5-tetrafluorobenzoic acid; PO, propylene oxide; TrFE, 3,3,3-trifluoropropylene oxide; IPA, isopropyl alcohol; TrFPA, 1,1,1-trifluoro-2-propanol.

Model reactions were designed as four matched comparisons in which one fluorination-related variable was changed while the reaction partner was held constant, thereby separating contributions that are coupled during ROAC (Figure 5).

First, PPA-mediated opening of PA and FPA compared the susceptibility of the two anhydrides toward a common alcohol-derived alkoxide. PPA was selected as a readily monitored, nonfluorinated primary-alcohol model whose phenyl group is remote from the reactive hydroxy group, allowing the effect of anhydride fluorination to be examined independently. At 100 °C, PPA gave approximately 63% FPA conversion after 1 h, compared with approximately 40% for PA; FPA conversion further reached approximately 95% after 2 h (Figure 5a). Consistent with this result, Fornacon-Wood et al. independently reported faster FPA/PO than PA/PO ring-opening copolymerization and exclusive FPA incorporation before PA in mixed-anhydride polymerizations, which they attributed to the higher electrophilicity imparted by the electron-withdrawing fluorine substituents [49]. Second, DO opening mediated by BzA or FBzA was used to evaluate how fluorination of an aromatic carboxylate affects epoxide-opening reactivity. BzA and FBzA were selected as structurally matched small-molecule surrogates for the nonfluorinated and fluorinated aromatic carboxylate chain ends, respectively, while DO served as a common nonfluorinated, low-volatility epoxide so that only the carboxylate structure was varied. At 60 °C, the FBzA–DO system reached approximately 45% conversion within the first few hours, whereas the BzA–DO system reached only approximately 37% even after approximately 65 h (Figure 5b).

Third, BzA-mediated opening of PO and TrFE isolated the effect of epoxide fluorination on electrophilic reactivity. PO and TrFE were selected as a matched CH_3_/CF_3_-substituted epoxide pair, while BzA provided the same aromatic carboxylate partner in both reactions. At 100 °C, TrFE reached approximately 95% conversion after approximately 35 h, while PO conversion was approximately 19% at the same reaction time (Figure 5c). Finally, PA opening by IPA or TrFPA evaluated how fluorination affects alcohol/alkoxide nucleophilicity. IPA and TrFPA form a matched pair in which one CH_3_ group is replaced by CF_3_, while PA was held constant as the common anhydride electrophile. At 60 °C, PA conversions after 1.5 h were approximately 72% and 32% with IPA and TrFPA, respectively, consistent with lower nucleophilicity in the fluorinated alcohol/alkoxide model system (Figure 5d). Because the matched pairs were sampled discretely and different matched pairs were measured at different temperatures, these datasets are used only for qualitative comparisons within each pair. All measured points are shown, and the lines are visual guides rather than kinetic fits.

Together, these matched comparisons indicate that fluorination does not exert a uniform effect throughout the tested reaction cycle. Fluorination increased the reactivity of the tested cyclic electrophiles, was associated with lower nucleophilicity in the fluorinated alcohol/alkoxide model system, and increased the observed epoxide-opening reactivity in the fluorinated aromatic carboxylate model system. For the FPA/PA competition, faster engagement of FPA and the higher epoxide-opening reactivity observed in the fluorinated aromatic carboxylate model system act in the same direction, providing a plausible mechanistic rationale for formation of the FPA-derived central segment before PA incorporation.

### 3.4. Thermal Characterization

The basic thermal properties of the three block copolyesters were evaluated by DSC and TGA (Figure S4). F100–N100, F150–N50, and F50–N150 exhibited glass-transition temperatures of 41, 43, and 39 °C, respectively, indicating only a minor dependence on the FPA:PA ratio over the investigated compositions. Their 5% weight-loss temperatures were 320, 300, and 295 °C, respectively, demonstrating good thermal stability for all three samples.

## 4. Conclusions

Fluorination generated sufficient kinetic contrast between the structurally matched anhydrides FPA and PA to produce block polyesters directly from a common monomer mixture. FPA was consumed to >99% conversion before PA incorporation, and bidirectional initiation placed poly(FPA-*alt*-PFE) at the chain center before growth of the two poly(PA-*alt*-PFE) outer segments. Conversion-resolved NMR, SEC evolution, end-group analysis, and DOSY NMR collectively supported covalent block formation, while the same incorporation order was retained across three FPA:PA feed ratios. BSL analysis consistently placed all three feed compositions in the real-block regime, with fitted effective descriptors of $r_{FPA}\approx 8.3–8.6\times 10^{2}$ and $r_{PA}\approx 1.2\times 10^{-3}$. Matched model reactions further indicated that the observed hierarchy is consistent with coupled effects of fluorination on anhydride ring-opening and the observed epoxide-opening reactivity of the corresponding aromatic carboxylate model system. These results identify H-to-F substitution on a matched aromatic anhydride scaffold as a practical kinetic design variable for programming block sequence within a single ROAC manifold, while providing a controlled structural comparison for evaluating fluorination-induced reactivity differences. Beyond the present kinetic analysis, the ability to vary the fluorinated block composition within a common polymerization platform provides a basis for future studies connecting programmed sequence and architecture with the phase behavior and functional properties of fluoropolymer materials.

## Data Availability

The original contributions presented in this study are included in the article and the Supporting Information. Further inquiries can be directed to the corresponding authors.

## Supplementary Materials

The following supporting information can be downloaded at https://www.mdpi.com/article/10.3390/polym18161977/s1, Section S1, additional results of the Beckingham–Sanoja–Lynd nonterminal analysis (Table S1); Section S2, supplementary data treatment for the model reactions; Section S3, polymerization and characterization data (Tables S2 and S3 and Figures S1–S4); and Section S4, reagent charges for the matched model reactions (Table S4).
