# Supplementary material for: Fluorine-Modulated Reactivity Enables One-Pot Kinetic Sequence Programming of Block Copolyesters
Source: Polymers (Basel). 2026 Aug 14;18(16):1977. doi: 10.3390/polym18161977 (PMC13516752; doi:10.3390/polym18161977)
Supplement: Supplementary file 1 [file polymers-18-01977-s001.zip › polymers-4483996-supplementary.pdf]

Supporting Information

Fluorine-Modulated Reactivity Enables One-Pot Kinetic Sequence Programming of Block Copolyesters

Chun-Yao Ke [1,2], Ryota Suzuki [3], Takuya Yamamoto [3], Takuya Isono [3,*], Guey-Sheng Liou [1,*] and Toshifumi Satoh [3,4,5,*]

[1] C.-Y. Ke, Prof. G.-S. Liou
Institute of Polymer Science and Engineering
National Taiwan University
No. 1, Sec. 4, Roosevelt Road, Taipei 10617, Taiwan
E-mail: gsliou@ntu.edu.tw

[2] C.-Y. Ke
Graduate School of Chemical Sciences and Engineering
Hokkaido University
Sapporo 060-8628, Japan

[3] Dr. R. Suzuki, Prof. T. Yamamoto, Prof. T. Isono, Prof. T. Satoh
Division of Applied Chemistry, Faculty of Engineering
Hokkaido University
Sapporo 060-8628, Japan
E-mail: isono.t@eng.hokudai.ac.jp; satoh@eng.hokudai.ac.jp

[4] Prof. T. Satoh
List Sustainable Digital Transformation Catalyst Collaboration Research Platform
Institute for Chemical Reaction Design and Discovery
Hokkaido University
Sapporo 001-0021, Japan

[5] Prof. T. Satoh
Department of Chemical and Materials Engineering
National Central University
No. 300, Zhongda Road, Zhongli District, Taoyuan 320317, Taiwan

S1. Additional Results of the Beckingham–Sanoja–Lynd Nonterminal Analysis

The BSL nonterminal analysis was performed as described in Ref. [S1], with the definitions, integrated expressions, and fitting procedure provided in Section 2.5 of the main manuscript. The compositional-drift traces obtained at initial FPA:PA feed ratios of 100:100, 150:50, and 50:150 were fitted independently by nonlinear least squares. The fitted effective reactivity ratios and corresponding sequence classifications are summarized in Table S1.

Table S1. Effective reactivity ratios obtained from independent BSL fitting.

Initial FPA:PA feed	r_{FPA}	r_{PA}	$r_{FPA} \times r_{PA}$	Sequence regime
100:100 (F100–N100)	850.3	0.0012	1.020	Real-block
150:50 (F150–N50)	831.2	≈0.0012	≈0.997	Real-block
50:150 (F50–N150)	858.6	≈0.0012	≈1.030	Real-block

The close agreement among the fitted r_{FPA} values supports the internal consistency of the effective nonterminal treatment. These values describe the extreme compositional drift and should be interpreted primarily according to their order of magnitude and corresponding sequence regime rather than as precisely determined elementary propagation-rate constants.

S2. Supplementary Data Treatment for the Model Reactions

S2.1 Conversion calculations

The model-reaction design, general conditions, temperatures, and NMR sampling procedure are described in the main manuscript. For epoxide ring-opening reactions, conversion was calculated from a diagnostic signal of the ring-opened product and the corresponding signal of the remaining cyclic epoxide:

$$P_{epoxide} = I_{opened} / \left(I_{opened} + I_{epoxide}\right)$$

where $I_{(opened)}$ is the integral of the ring-opened epoxide signal and $I_{(epoxide)}$ is the integral of the cyclic epoxide signal.

For anhydride ring-opening systems, the conversion was determined from the relative integrals of the ring-opened and cyclic aromatic signals. For PA, the cyclic anhydride signals appear at approximately 7.8–8.0 ppm and shift to 7.6–7.8 ppm after ring opening:

$$P_{anhydride} = I_{opened}/(I_{opened} + I_{cyclic})$$

where $I_{(cyclic)}$ corresponds to the cyclic anhydride aromatic region (7.8 to 8.0 ppm for PA) and $I_{(opened)}$ corresponds to the ring-opened aromatic region (7.6 to 7.8 ppm for PA). The same approach was applied to the other anhydride analogs using their assigned diagnostic resonances.

S2.2 Model-reaction data presentation and interpretation limits

All measured conversion points are presented in Figure 5, and no data points were excluded. The lines serve only as visual guides between the measured points and do not represent least-squares or kinetic fits. Because the model reactions were designed as qualitative matched-pair comparisons rather than replicate kinetic measurements, the data were not used to obtain statistical fitting parameters or elementary rate constants.

The model reactions provide mechanistic support rather than a complete kinetic description of the polymerization. In particular, BzA and FBzA are small-molecule aromatic carboxylic-acid model compounds and do not reproduce the complete structure, ion-pairing environment, or conformational constraints of the propagating chain ends generated from PA and FPA.

S3. Polymerization and Characterization Data

Table S2. Summary of the one-pot polymerizations and final polymer characterization.

Sample	FPA:PA feed	Final time (h)	FPA conv. (%)	PA conv. (%)	$M_{n,SEC}$ (kg mol^{-1})	$Đ$	Yield (%)
F100–N100	100:100	31	100	99	17.7	1.08	95
F150–N50	150:50	15	100	99	12.0	1.08	93
F50–N150	50:150	30	100	98	49.7	1.12	95

All reactions used [BDM]0/[CsOPiv]0/[total anhydride]0/[PFE]0 = 1:0.5:200:400. Conversions and final SEC values are those reported in the corresponding time-resolved datasets and figures.

Table S3. Reagent charges used for the three one-pot polymerizations.

Component	F100–N100	F150–N50	F50–N150
CsOPiv	11.70 mg (0.050 mmol)	11.70 mg (0.050 mmol)	11.70 mg (0.050 mmol)
BDM	13.92 mg (0.100 mmol)	13.92 mg (0.100 mmol)	13.92 mg (0.100 mmol)
FPA	2200.8 mg (10.0 mmol)	3301.2 mg (15.0 mmol)	1100.4 mg (5.0 mmol)
PA	1481.2 mg (10.0 mmol)	740.6 mg (5.0 mmol)	2221.8 mg (15.0 mmol)
PFE	15044.8 mg (40.0 mmol)	15044.8 mg (40.0 mmol)	15044.8 mg (40.0 mmol)

PFE served as both comonomer and reaction medium. After formation of the FPA-rich segment, the reaction mixture remained homogeneous at 120 °C but became highly viscous. Dry THF (1.0 mL) was therefore added to maintain effective stirring during the subsequent PA–PFE growth stage. The polymer-rich mixture became heterogeneous only upon cooling. The products were diluted with THF, precipitated into cold methanol, purified by preparative SEC, and dried under vacuum at 80 °C.

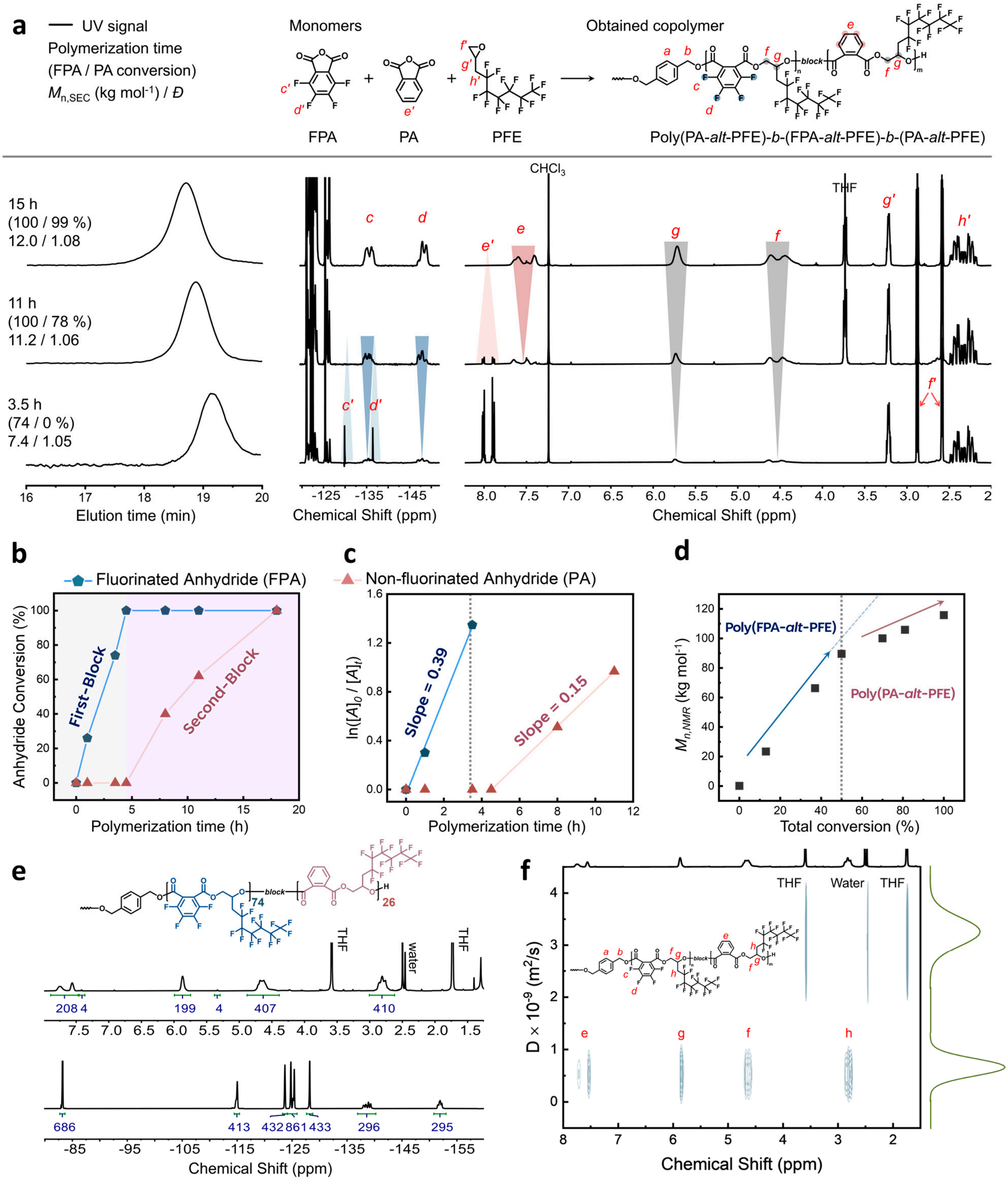

Figure S1. Synthesis and characterization of F150–N50. (a) Time-resolved SEC, ^{19}F NMR, and ^{1}H NMR data. (b) Anhydride conversion versus time. (c) Pseudo-first-order plots. (d) $M_{n,NMR}$ versus total anhydride conversion. (e) ^{1}H NMR spectrum of the purified copolymer. (f) DOSY NMR spectrum. Conditions: $[BDM]_0/[CsOPiv]_0/[FPA]_0/[PA]_0/[PFE]_0$ = 1:0.5:150:50:400. SEC eluent: THF; UV detection wavelength: 280 nm.

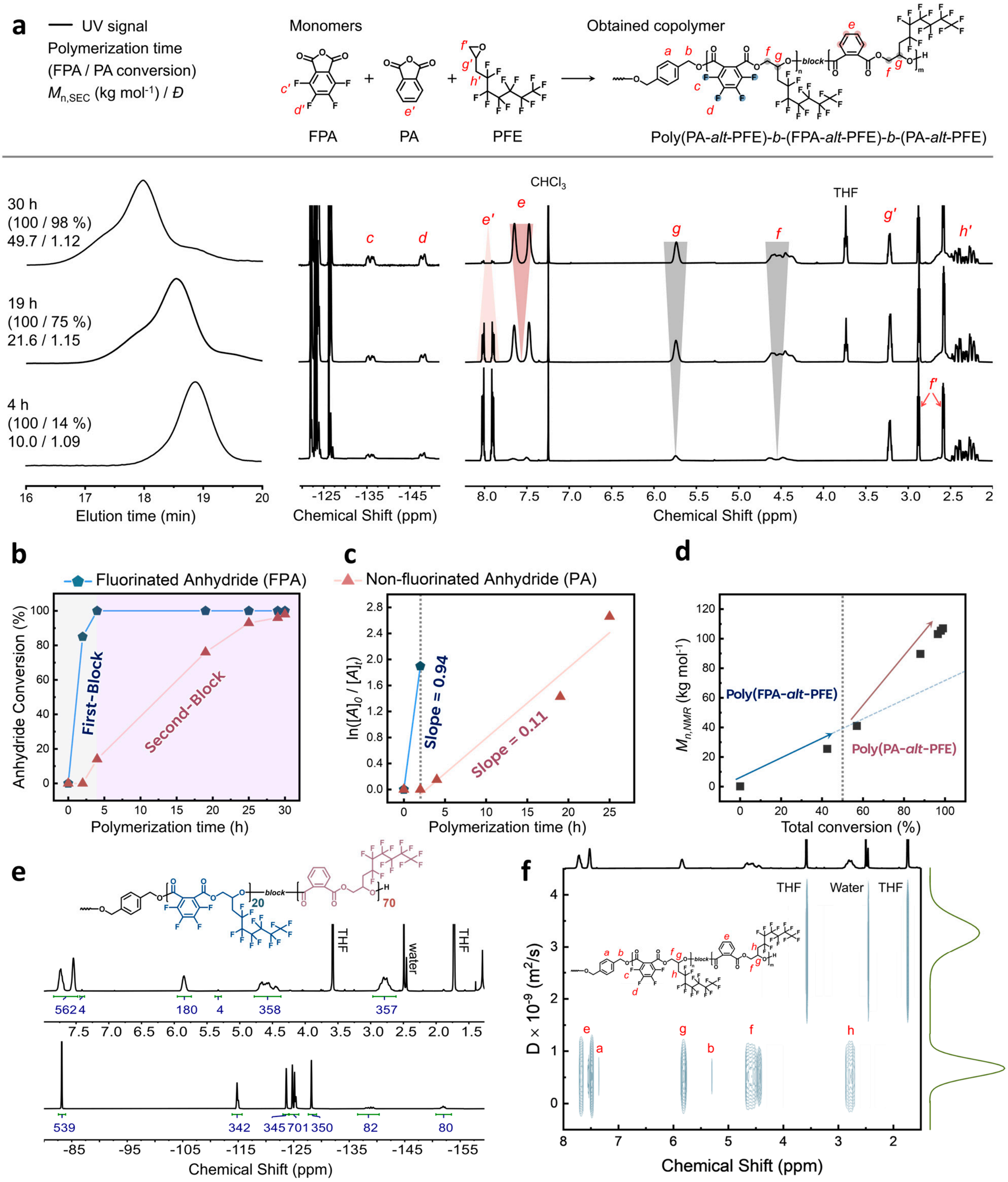

Figure S2. Synthesis and characterization of F50–N150. (a) Time-resolved SEC, ^{19}F NMR, and ^{1}H NMR data. (b) Anhydride conversion versus time. (c) Pseudo-first-order plots. (d) $M_{n,NMR}$ versus total anhydride conversion. (e) ^{1}H NMR spectrum of the purified copolymer. (f) DOSY NMR spectrum. Conditions: $[BDM]_0/[CsOPiv]_0/[FPA]_0/[PA]_0/[PFE]_0$ = 1:0.5:50:150:400. SEC eluent: THF; UV detection wavelength: 280 nm.

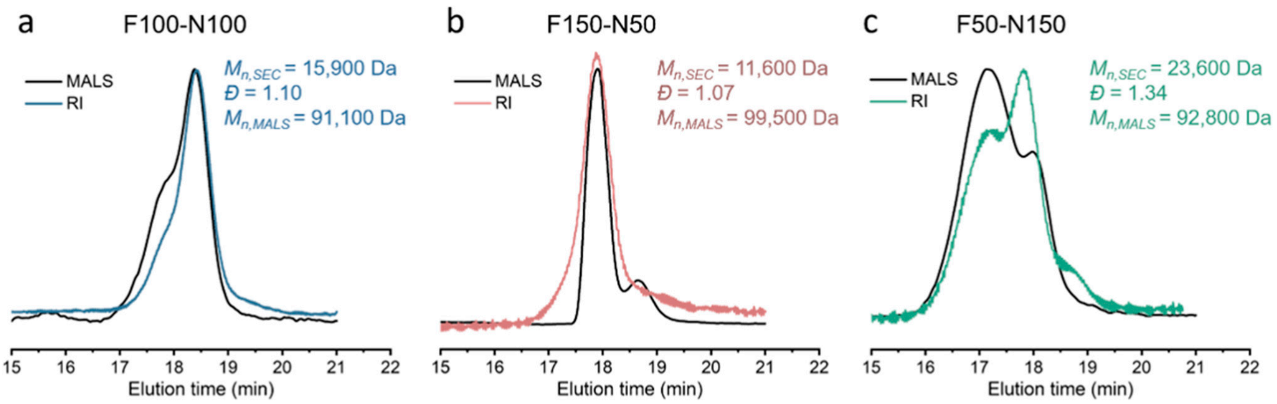

d

Sample	F100-N100	F150-N50	F50-N150
$M_{n,NMR}$ (Da)	121,500	115,500	97,200
$M_{n,SEC}$ (Da)	15,900	11,600	23,600
$M_{n,MALS}$ (Da)	91,100	99,500	92,800
$M_{n,Theoretical}$ (Da)	112,000	115,600	108,400
$Đ_{MALS}$	1.10	1.07	1.34
dn/dc (mL g^{-1})	0.011	0.018	0.013

Figure S3. Absolute molar-mass determination of the fluorinated block copolyesters by SEC-MALS. (a–c) MALS and RI detector traces of F100–N100, F150–N50, and F50–N150, respectively. (d) Summary of the NMR-estimated, polystyrene-calibrated SEC, SEC-MALS absolute, and theoretical number-average molar masses, together with $Đ_{MALS}$ and dn/dc values. The $M_{n,SEC}$ values shown here were obtained from the $CHCl_3$ SEC-MALS measurements.

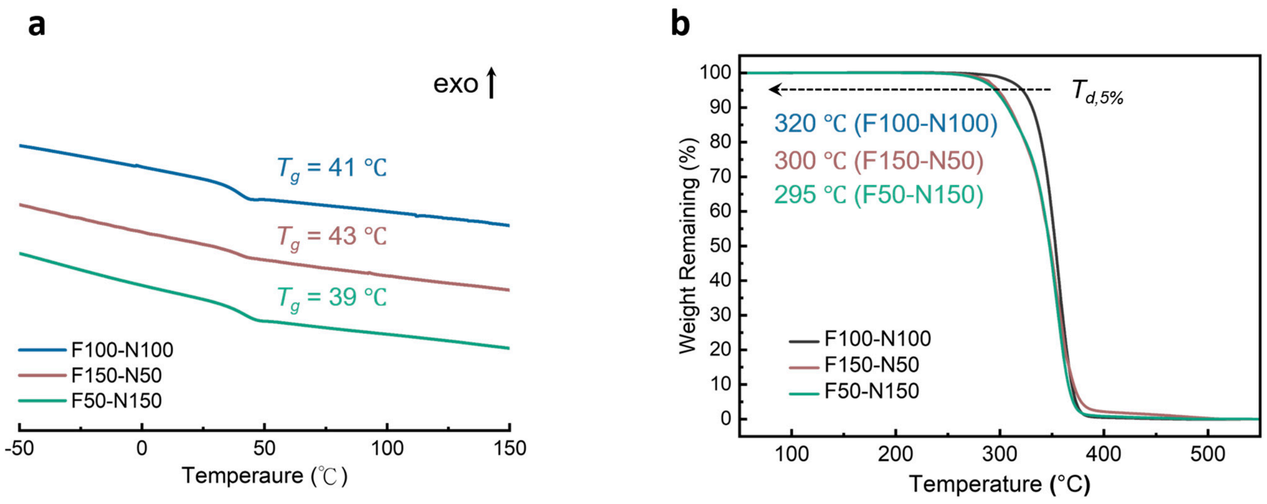

Figure S4. Thermal characterization of the block copolyesters. (a) DSC second-heating traces of F100–N100, F150–N50, and F50–N150 recorded under nitrogen at 10 °C min^{-1}. (b) TGA curves recorded under nitrogen at 10 °C min^{-1}. The corresponding glass-transition temperatures (T_g) and 5% weight-loss temperatures ($T_{d,5\%}$) are indicated.

S4. Reagent Charges for the Matched Model Reactions

Table S4. Reagent charges used for the matched model reactions.

Entry	Substrate 1	Substrate 2	CsOPiv	THF	Temperature
1a	PPA, 136.2 mg (1.00 mmol)	PA, 148.1 mg (1.00 mmol)	46.8 mg (0.200 mmol)	1.0 mL	100 °C
1b	PPA, 136.2 mg (1.00 mmol)	FPA, 220.1 mg (1.00 mmol)	46.8 mg (0.200 mmol)	1.0 mL	100 °C
2a	BzA, 122.1 mg (1.00 mmol)	DO, 156.3 mg (1.00 mmol)	46.8 mg (0.200 mmol)	1.0 mL	60 °C
2b	FBzA, 194.1 mg (1.00 mmol)	DO, 156.3 mg (1.00 mmol)	46.8 mg (0.200 mmol)	1.0 mL	60 °C
3a	BzA, 122.1 mg (1.00 mmol)	PO, 58.1 mg (1.00 mmol)	46.8 mg (0.200 mmol)	1.0 mL	100 °C
3b	BzA, 122.1 mg (1.00 mmol)	TrFE, 112.1 mg (1.00 mmol)	46.8 mg (0.200 mmol)	1.0 mL	100 °C
4a	IPA, 60.1 mg (1.00 mmol)	PA, 148.1 mg (1.00 mmol)	46.8 mg (0.200 mmol)	1.0 mL	60 °C
4b	TrFPA, 114.1 mg (1.00 mmol)	PA, 148.1 mg (1.00 mmol)	46.8 mg (0.200 mmol)	1.0 mL	60 °C

All reactions were conducted in pre-dried sealed vessels under argon using substrate 1/substrate 2/CsOPiv = 1:1:0.2. Each organic substrate was used at an initial concentration of approximately 1.0 M.

S5. Supporting References

[S1] Beckingham, B.S.; Sanoja, G.E.; Lynd, N.A. Simple and accurate determination of reactivity ratios using a nonterminal model of chain copolymerization. *Macromolecules* **2015**, 48, 6922–6930.
